# Genomic Profiling Identified *ERCC2* E606Q Mutation in Helicase Domain Respond to Platinum-Based Neoadjuvant Therapy in Urothelial Bladder Cancer

**DOI:** 10.3389/fonc.2020.01643

**Published:** 2020-08-26

**Authors:** Yosuke Hirotsu, Hitoshi Yokoyama, Kenji Amemiya, Takashi Hagimoto, Kyoko Hosaka, Toshio Oyama, Hitoshi Mochizuki, Masao Omata

**Affiliations:** ^1^Genome Analysis Center, Yamanashi Central Hospital, Kofu, Japan; ^2^Department of Urology, Yamanashi Central Hospital, Kofu, Japan; ^3^Department of Pathology, Yamanashi Central Hospital, Kofu, Japan; ^4^Department of Gastroenterology, Yamanashi Central Hospital, Kofu, Japan; ^5^The University of Tokyo, Bunkyo-ku, Japan

**Keywords:** bladder cancer, NGS, ERCC2, actionable mutation, precision medicine

## Abstract

Genomic profiling of tumors enables therapeutic decisions, and identifying drug-matched mutations will prolong survival and prognosis. Here, we generated a custom panel for detecting genetic alterations in 19 patients with urothelial bladder cancer. This panel targeted 71 genes associated with urological cancer. Targeted sequencing was performed on formalin-fixed paraffin-embedded tumor tissues. Paired patient-matched tumor and blood samples were subjected to this analysis. A total of 142 somatic mutations were detected in 19 tumor tissues. At least one non-synonymous mutation was detected in all tumor tissues, and *KDM6A, KMT2D, TP53*, *KMT2C*, *PIK3CA*, and *ERCC2* were recurrently mutated. Chromatin remodeling and epigenetic modifier genes are frequently mutated. Of 142 mutations, 69 mutations (49%) were annotated to have oncogenic potential. Furthermore, 74% of patients were expected to receive targeted therapy due to drug-matched mutations being identified in their tumors. Among this cohort, a patient harbored an *ERCC2* helicase domain mutation and would be expected to respond to platinum-based therapy. As expected, the patient received carboplatin-containing neoadjuvant therapy with a remarkable response. Furthermore, tumor-derived mutations in urine were rapidly decreased after neoadjuvant therapy. These results suggested targeted sequencing could help to detect drug-matched somatic mutations and indicate single or combination therapy for cancer patients.

## Introduction

Bladder cancer (BC) is one of the most common cancers, and more than 90% of BC cases are urothelial carcinoma. Urothelial BC comprises squamous cell, adenocarcinoma, and other rarer types (1). BC is among the most prevalent cancers worldwide, with approximately 430,000 new diagnoses each year (2). Cigarette smoking is the most prominent risk factor and contributes to approximately 50% of BC cases (3). Other risk factors are known including petrochemical and other industrial compound exposure and bladder stones.

Approximately 75% of newly diagnosed cases are non-muscle-invasive BC (NMIBC) and 25% cases are muscle-invasive BC (MIBC) (4). Patients with MIBC have a worse prognosis than those with NMIBC, owing to the development of metastatic disease in 50% of patients with MIBC. Therapy for NMIBC consists of transurethral resection of the bladder tumor (TURBT), which is followed by intravesical administration of bacillus Calmette-Guerin (BCG) or intravesical chemotherapy. The majority of NMIBC patients will recur after complete resection, therefore, long-term surveillance is mandatory for such patients. The most common therapy for MIBC is radical cystectomy, followed by chemoradiotherapy. The use of neoadjuvant platinum-based combination chemotherapy is considered as standard of care. Long-term follow-up is needed for clinical management to survey tumor recurrence and metastases.

Comprehensive genomic analysis revealed the driver genes for urothelial BC (5). Across several types of tumors, the tumor mutational burden is relatively high in urothelial BC, followed by melanoma and lung cancer (5). The Cancer Genome Atlas (TCGA) identified potential molecular targets in the PI3K–AKT–mTOR and receptor tyrosine kinase (RTK)–MAPK pathways in patients with MIBC. Epigenetic and chromatin remodeling factors are also disrupted at a high frequency. Furthermore, DNA damage response genes (*ERCC2*, *ATM*, *RB1*, and *FANCC*) and *ERBB2* are biomarkers for predicting response to neoadjuvant chemotherapy (6, 7).

These genetic features aid the identification of frequently mutated genes (8), which are expected to be therapeutic targets and thus improve patient prognosis (9). Mutational profiles can reveal drug-matched mutations and be useful for selecting appropriate treatment for cancer patients in the precision medicine era (10, 11). In this study, to examine the genomic profiles in urothelial BC, we generated a customized panel to investigate a set of significantly mutated genes associated with urological cancers based on TCGA and other reports. We examined these genetic alterations as potential therapeutic targets that respond to neoadjuvant therapy.

## Materials and Methods

### Patients and DNA Extraction

We studied 19 patients who were diagnosed with urothelial BC who received TURBT (13 NMIBC and six MIBC; 18 males; and 3 females). Informed consent was obtained from all patients. This study was approved by the Institutional Review Board of clinical research and genome research committee at Yamanashi Central Hospital (G-2018-1) and complied with Declaration of Helsinki principles. Peripheral blood samples were obtained and centrifuged to separate buffy coats (12). Urine was also centrifuged and separate into urine precipitate and supernatant. Buffy coat, urine precipitate, and urine supernatants were stored at −80°C until DNA extraction. Buffy coat was used as a control to detect somatic mutations in tumor tissues. DNA from buffy coat and urine precipitate was extracted with the QIAamp DNA Blood Mini QIAcube Kit (Qiagen, Hilden, Germany) and the DNA concentration was determined using a Nano Drop 2000 (Thermo Fisher Scientific, Waltham, MA, United States). DNA was extracted from the urine supernatant with the MagMax Cell Free DNA extraction kit and KingFisher Duo Prime (Thermo Fisher Scientific). DNA from urine supernatant was determined using the Qubit dsDNA HS Assay Kit and Qubit 3.0 fluorometer (Thermo Fisher Scientific) according to the manufacturer’s instructions.

### Tumor Sample Preparation and Laser Capture Microdissection

Tumor tissues were resected by TURBT and fixed using 10% buffered formalin (13). Serial 10 μm sections were prepared from formalin-fixed paraffin-embedded tissues (FFPE). Sections were stained with haematoxylin-eosin and reviewed by a pathologist to check the tumor location. To enrich the tumor content, laser capture microdissection was performed as described previously (14). Tumor DNA was extracted using the GeneRead DNA FFPE Kit (Qiagen). The FFPE DNA quality was determined by quantitative real-time PCR with two different primers targeting *RNase P* genes, as described previously (15).

### Gene Selection and Targeted Sequencing

We searched TCGA data and literature (5, 16–22) and selected 71 genes related to urological cancer (kidney cancer, prostate cancer, and BC) as described previously (23). A total of 3,652 primer pairs were designed by Ion AmpliSeq Designer contained within this panel (covering 365.34 kb) (23). Library preparation was conducted by amplicon-based multiplex PCR with Ion AmpliSeq Plus Kit (Thermo Fisher Scientific) as described previously (24–26). The library concentration was determined by quantitative real-time PCR using an Ion Library Quantitation Kit. Emulsion PCR and chip loading were performed on the Ion Chef with the Ion PI Hi-Q Chef kit. Sequencing was performed on the Ion Proton Sequencer (Thermo Fisher Scientific). Sequence data analysis was performed as described previously (24).

To evaluate the oncogenicity of mutations, we searched the OncoKB database (27). All identified mutations in the tumors were analyzed. The “likely oncogenic” and “oncogenic” mutations in the OncoKB database were denoted as oncogenic mutations. We also annotated the actionable mutations using OncoKB and cBioPortal (28).

### *In silico* Analysis

To examine the impact of mutations on the protein function, three *in silico* tools were utilized. SIFT predicts whether an amino acid substitution affects protein function based on sequence homology and the physical properties of amino acids (29). Mutation with a SIFT score between 0 and 0.05 is predicted to be deleterious and between 0.05 and 1.0 is predicted to be benign. PolyPhen-2 (Polymorphism Phenotyping v2) is a tool that predicts the possible impact of an amino acid substitution on the structure and function of a human protein using straightforward physical and comparative considerations (30). Mutation with a PolyPhen score between 0 and 0.15 is predicted to be benign, between 0.15 and 1.0 is possibly damaging, and between 0.85 and 1.0 is damaging. SIFT and PolyPhen score was used from the Ion Reporter analysis. CADD (Combined Annotation Dependent Depletion) can be used to predict the mutational impact across the genome-wide distribution of scores for all 9 billion potential single nucleotide variants (SNV) (31, 32). CADD phred scores are normalized to all potential variants. The bottom 90% (approximately 7.7 billion) of SNVs (approximately 8.6 billion) are compressed into scaled CADD phred scores between 0 to 10, while the next 9% (top 10% to top 1%) was between 10 and 20, and top 1% was above 20 (31, 33). We also surveyed somatic mutations reported in the Catalog of Somatic Mutations in Cancer (COSMIC) database referring to the mutations in *ERCC2* (RefSeq ID: NM_000400.3) (34).

## Results

### Somatic Mutations in Primary Tumors

Nineteen patients with urothelial BC [13 NMIBC (Case #1–13) and six MIBC (Case #14–19)] were analyzed in this study. All tumor tissues were obtained by TURBT. We conducted laser-capture microdissection from FFPE tumor tissues to enrich the tumor purity. Targeted sequencing was performed using paired buffy coat and tumor tissue from each patient to detect somatic mutations. The sequencing panel for target sequencing covered 71 significantly mutated genes associated with urological cancer including urothelial bladder, kidney, and prostate cancer revealed by TCGA and other projects (23). We achieved sufficient sequence reads in the target region (mean, 95.7%) and sufficient coverage depth (mean, 1630-fold; Supplementary Table 1). As result, a total of 142 somatic mutations were identified in the primary urothelial bladder tumors (average, 7.5 mutations per tumor; range, 1–13).

**FIGURE 1:**
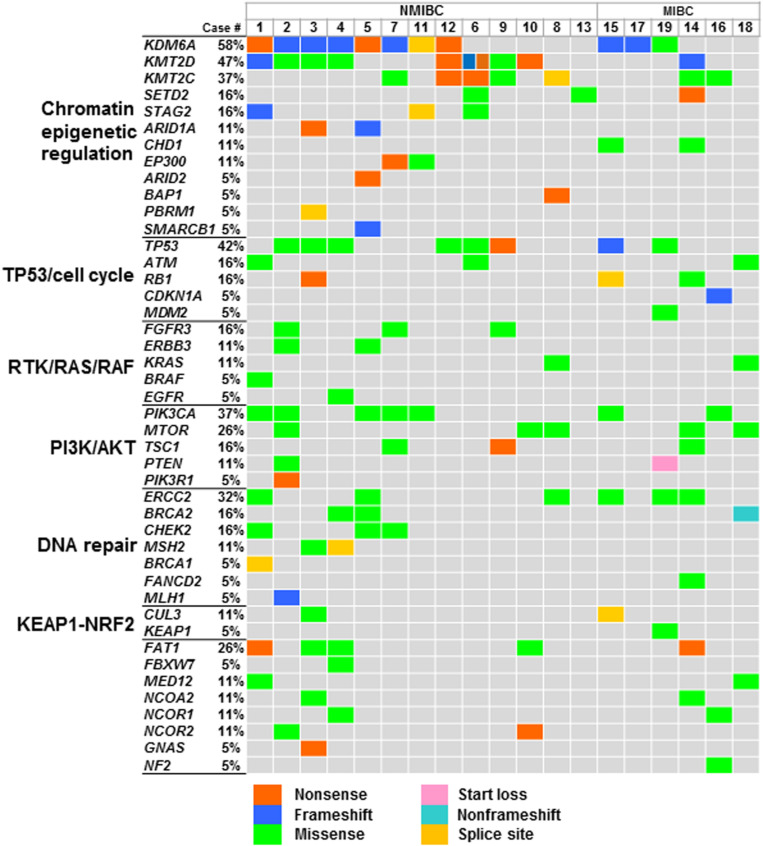
Genomic profiles in urothelial BC. Oncoprint showing the mutated genes identified in primary tumors; mutation types are colored. Signaling pathways of genes, gene symbols, and mutational frequency are shown on the left. Abbreviation: NMIBC, non-muscle-invasive bladder cancer; MIBC, muscle-invasive bladder cancer.

At least one non-synonymous mutation was identified in all primary tumors (Figure 1). Frequently mutated genes were *KDM6A* (58%; *n* = 11), *KMT2D* (also known as *MLL2*, 47%; *n* = 9), *TP53* (42%; *n* = 8), *KMT2C* (also known as *MLL3*, 37%; *n* = 7), *PIK3CA* (37%; *n* = 7), and *ERCC2* (32%; *n* = 6). Of note, truncating mutations in *KDM6A, KMT2D*, and *KMT2C* were frequently observed (74%; 14/19), suggesting that disruption of the epigenetic regulatory pathway may be involved in tumor development in urothelial BC (5).

### Determination of Oncogenic Mutations in Urothelial BC

We evaluated the oncogenicity of mutations using the OncoKB database (27). Of the 142 somatic mutations identified in the primary tumors, 69 mutations (49%) were oncogenic and at least one oncogenic mutation was identified in tumors from 95% of patients (18/19; Table 1). Of 69 oncogenic mutations, 13 were gain-of-function mutations and 56 were loss-of-functions. Gain-of-function mutations were found in *KRAS*, *BRAF*, *PIK3CA*, *ERBB3*, and *FGFR3* (Table 1). Loss-of-function mutations were observed in chromatin remodeling and epigenetic modifiers (*ARID1A*, *ARID2*, *KDM6A*, *KMT2C*, *KMT2D*, *SMARCB1*, *BAP1*, *EP300*, *PBRM1*, *SETD2*, and *STAG2*), DNA repair (*BRCA1*, *BRCA2*, *MLH1*, *MSH2*, and *ERCC2*), p53 cell cycle pathway (*TP53*, *RB1*, and *CDKN1A*), and PI3K–AKT–mTOR pathway (*PIK3R1*, *TSC1*). Genomic profiles identified 74% (14/19) of patients had actionable mutations that were expected to receive targeting therapy (Table 1 and Supplementary Table 2). Notably, multiple actionable mutations were identified in nine out of 19 patients who would benefit from combination therapy (10). These results showed panel-based genomic profiling is promising for selecting drug treatment.

**TABLE 1:** Oncogenic and actionable mutations.

Case #	Gene	Mutation	VAF	Oncogenecity	Function	Actionable mutation
Case 1	*KDM6A*	Q677*	0.75	Likely oncogenic	Likely loss of function	Yes
(NMIBC)	*ERCC2*	N238S	0.43	Likely oncogenic	Likely loss of function	Yes
	*PIK3CA*	E545K	0.42	Oncogenic	Gain of function	Yes
	*BRCA1*	splice site	0.38	Likely oncogenic	Likely loss of function	Yes
	*KMT2D*	D5198fs	0.38	Likely oncogenic	Likely loss of function	
	*FAT1*	E1675*	0.36	Likely oncogenic	Likely loss of function	
	*STAG2*	N780fs	0.23	Likely oncogenic	Likely loss of function	
	*BRAF*	K601N	0.1	Likely oncogenic	Gain of function	Yes
Case 2	*KDM6A*	Y215fs	0.82	Likely oncogenic	Likely loss of function	Yes
(NMIBC)	*MLH1*	E679fs	0.79	Likely oncogenic	Likely loss of function	Yes
	*FGFR3*	G380R	0.45	Likely oncogenic	Gain of function	
	*TP53*	G245S	0.42	Oncogenic	Loss of function	
	*PIK3R1*	Y452*	0.28	Likely oncogenic	Likely loss of function	
	*ERBB3*	V104M	0.09	Oncogenic	Gain of function	
	*TP53*	R196*	0.04	Oncogenic	Loss of function	
	*PIK3CA*	R108H	0.03	Likely oncogenic	Gain of function	Yes
Case 3	*KDM6A*	G514fs	0.94	Likely oncogenic	Likely loss of function	Yes
(NMIBC)	*TP53*	G245D	0.91	Oncogenic	Loss of function	
	*ARID1A*	W2050*	0.51	Likely oncogenic	Likely loss of function	
	*RB1*	S618*	0.36	Likely oncogenic	Likely loss of function	
	*PBRM1*	splice site	0.09	Likely oncogenic	Likely loss of function	
Case 4	*MSH2*	splice site	0.7	Likely oncogenic	Likely loss of function	Yes
(NMIBC)	*KDM6A*	R1054fs	0.48	Likely oncogenic	Likely loss of function	Yes
	*TP53*	C141S	0.37	Oncogenic	Loss of function	
	*TP53*	E286K	0.34	Oncogenic	Loss of function	
	*FBXW7*	R465C	0.33	Oncogenic	Loss of function	
Case 5	*KDM6A*	Q542*	0.9	Likely oncogenic	Likely loss of function	Yes
(NMIBC)	*SMARCB1*	T310fs	0.43	Likely oncogenic	Likely loss of function	Yes
	*PIK3CA*	E545K	0.41	Oncogenic	Gain of function	Yes
	*ARID2*	Q609*	0.39	Likely oncogenic	Likely loss of function	
	*ARID1A*	P729fs	0.26	Likely oncogenic	Likely loss of function	
Case 6	*KMT2C*	E2626*	0.46	Likely oncogenic	Likely loss of function	
(NMIBC)	*KMT2D*	R2771*	0.41	Likely oncogenic	Likely loss of function	
	*KMT2D*	Q3967fs	0.37	Likely oncogenic	Likely loss of function	
	*TP53*	R213Q	0.13	Oncogenic	Loss of function	
Case 7	*KDM6A*	E216fs	0.92	Likely oncogenic	Likely loss of function	Yes
(NMIBC)	*PIK3CA*	H1047R	0.48	Oncogenic	Gain of function	Yes
	*EP300*	E1525*	0.47	Likely oncogenic	Likely loss of function	
Case 8	*KRAS*	Q22K	0.63	Oncogenic	Gain of function	Yes
(NMIBC)	*ERCC2*	N238S	0.48	Likely oncogenic	Likely loss of function	Yes
	*BAP1*	Q253*	0.31	Likely oncogenic	Likely loss of function	
	*KMT2C*	splice site	0.22	Likely oncogenic	Likely loss of function	
Case 9	*TSC1*	Q830*	0.84	Likely oncogenic	Likely loss of function	Yes
(NMIBC)	*TP53*	Q331*	0.82	Likely oncogenic	Likely loss of function	
Case 10 (NMIBC)	*KMT2D*	S4073*	0.43	Likely oncogenic	Likely loss of function	
Case 11	*STAG2*	splice site	0.88	Likely oncogenic	Likely loss of function	
(NMIBC)	*KDM6A*	splice site	0.87	Likely oncogenic	Likely loss of function	Yes
	*PIK3CA*	N345K	0.43	Oncogenic	Gain of function	Yes
	*PIK3CA*	H1047R	0.17	Oncogenic	Gain of function	Yes
Case 12	*KDM6A*	S689*	0.81	Likely oncogenic	Likely loss of function	Yes
(NMIBC)	*KMT2D*	Q2902*	0.38	Likely oncogenic	Likely loss of function	
	*TP53*	F113L	0.1	Oncogenic	Loss of function	
	*KMT2C*	Y987*	0.06	Likely oncogenic	Likely loss of function	
	*TP53*	E271K	0.04	Oncogenic	Loss of function	
	*TP53*	K139N	0.04	Oncogenic	Loss of function	
	*TP53*	M246I	0.04	Oncogenic	Loss of function	
Case 13 (NMIBC)	ND	–	−	–	–	
Case 14	*SETD2*	Q1667*	0.39	Likely oncogenic	Likely loss of function	
(MIBC)	*FAT1*	Q1441*	0.37	Likely oncogenic	Likely loss of function	
	*KMT2D*	T176fs	0.15	Likely oncogenic	Likely loss of function	
Case 15	*KDM6A*	H1101fs	0.73	Likely oncogenic	Likely loss of function	Yes
(MIBC)	*RB1*	splice site	0.53	Likely oncogenic	Likely loss of function	
	*TP53*	G199fs	0.46	Oncogenic	Loss of function	
	*PIK3CA*	E545K	0.38	Oncogenic	Gain of function	Yes
Case 16	*PIK3CA*	E542V	0.38	Oncogenic	Gain of function	Yes
(MIBC)	*CDKN1A*	C41fs	0.29	Likely oncogenic	Likely loss of function	
Case 17 (MIBC)	*KDM6A*	T1125fs	0.83	Likely oncogenic	Likely loss of function	Yes
Case 18	*BRCA2*	R2666_S2667del	0.38	Likely oncogenic	Likely loss of function	Yes
(MIBC)	*KRAS*	G12R	0.07	Oncogenic	Gain of function	Yes
Case 19 (MIBC)	*TP53*	H178D	0.57	Oncogenic	Loss of function	

ND, not detected; VAF, variant allele fraction; NMIBC, non-muscle-invasive BC; and MIBC, uscle-invasive BC. Asterisk (*) means truncating mutation.

### Patient With *ERCC2* Mutation Responded to Platinum-Based Chemotherapy

**FIGURE 2:**
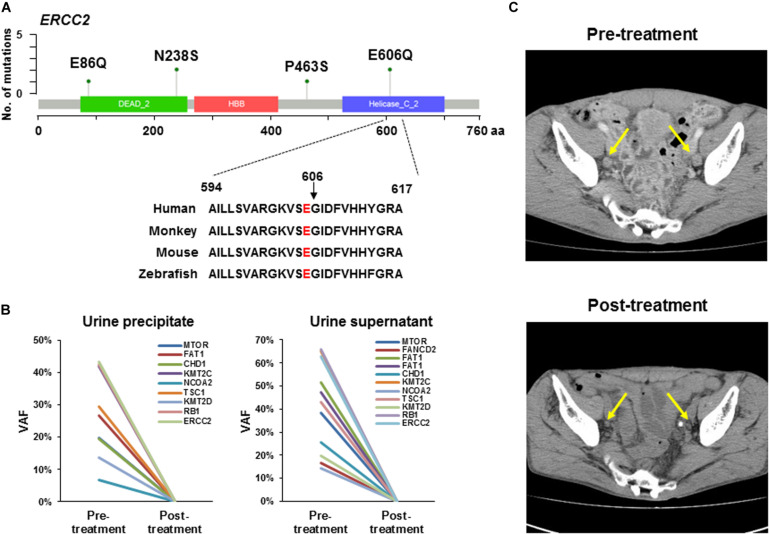
*ERCC2* mutations sensitive to platinum-based therapy. **(A)** Lollipop plots showing the mutation sites on *ERCC2*. Green circles indicate missense mutations. We identified four types of mutation (p.E86Q, p.N238S, p.P463S, and p.E606Q) in six patients. The amino acid is conserved at codon 606 across several species. **(B)** Tumor-identical mutations in urine precipitate (left panel) and supernatant (right panel) in Case #14. Variant allele fraction (VAF) was plotted before and after neoadjuvant therapy. All mutations in urine precipitation (left) and supernatant (right) were not detectable after chemotherapy. **(C)** Computed tomography image of lymph node metastasis in a patient (Case #14) harboring *ERCC2* p.E606Q mutation. This patient was responsive to carboplatin-based chemotherapy. Yellow arrows indicate lymph node metastasis. The metastatic tumor burden was decreased after platinum-based neoadjuvant chemotherapy.

A previous study showed a tumor with a defect in the nucleotide excision repair gene *ERCC2* was sensitive to platinum-based chemotherapy in MIBC and had a better prognosis (35). We found a total of six mutations in *ERCC2* including p.E606Q (*n* = 2), p.N238S (*n* = 2), p.E86Q (*n* = 1), and p.P463S (*n* = 1; Figure 2A and Table 2). To examine the pathogenicity of these mutants, we performed *in silico* analysis using SIFT, PolyPhen2, and CADD and defined all mutations as pathogenic mutations (Table 2).

**TABLE 2:** Mutation impact on protein function predicted by *in silico* analysis.

Case #	Gene	Mutation	Coding	SIFT (score)	PolyPhen2 (score)	CADD phred score	COSMIC
Case 1 (NMIBC)	*ERCC2*	p.N238S	c.713A > G	Deleterious (0)	Damaging (0.994)	25.5	COSM418170
Case 8 (NMIBC)	*ERCC2*	p.N238S	c.713A > G	Deleterious (0)	Damaging (0.994)	25.5	COSM418170
Case 5 (NMIBC)	*ERCC2*	p.P463S	c.1387C > T	Deleterious (0)	Damaging (0.998)	27.4	COSM1756994
Case 14 (MIBC)	*ERCC2*	p.E606Q	c.1816G > C	Deleterious (0)	Damaging (1)	25.7	NA
Case 15 (MIBC)	*ERCC2*	p.E86Q	c.256G > C	Deleterious (0)	Damaging (0.998)	25.4	COSM1304773
Case 19 (MIBC)	*ERCC2*	p.E606Q	c.1816G > C	Deleterious (0)	Damaging (1)	25.7	NA

*NA, not available.*

#### Case #14

The patient (Case #14) was diagnosed with MIBC and had bilateral pelvic lymph node metastases. In this tumor, *ERCC2* p.E606Q was identified, which is located in the DNA helicase domain and is conserved across species from human to zebrafish (Figure 2A). However, the oncogenicity of *ERCC2* p.E606Q was unclear from the OncoKB database. In a previous study, an *in vitro* assay showed *ERCC2* p.E606G impaired protein function (36). Although our identified mutation is a different amino acid change, we reasoned that this mutant was likely to be functionally defected and sensitive to platinum-based chemotherapy. Following this idea, this patient was treated with neoadjuvant therapy with carboplatin and gemcitabine (4 courses) before radical cystectomy. Pathological examination diagnosed tumor obtained by TURBT as high grade and muscle invasive urothelial carcinoma (*c*-stage IVa, cT4bN1M0). After cystectomy, resected tumor was high grade and muscle invasive urothelial carcinoma (*p*-stage IIIa, pT2aN0M0) by pathological review.

To investigate the treatment effect of chemotherapy, we examined whether the tumor-identical mutations were shed into the urine precipitation and supernatant by targeted sequencing. Nine and 11 tumor-identical mutations were identified in urine precipitate and supernatant before neoadjuvant chemotherapy, respectively (Figure 2B). However, all these mutations were not detectable after chemotherapy (Figure 2B). After neoadjuvant therapy, the primary tumor in the bladder and pelvic lymph node metastases had remarkably shrunk, as observed by computerized tomography (CT; Figure 2C). Furthermore, viable tumor cells were not observed in specimens from lymph node dissection, as determined by histological examination. The patient did not experience recurrence 1.5 year after radical cystectomy.

#### Case #19

The patient (Case #19) was diagnosed with MIBC and had metastases in right obturator lymph node and left external iliac nodes. The tumor also harbored *ERCC2* p.E606Q as the same with Case #14 aforementioned. Pathological diagnosis was high grade and muscle invasive urothelial carcinoma (cT2bN2M0). The patient received neoadjuvant chemotherapy with cisplatin and gemcitabine (3 courses) before cystectomy. After chemotherapy, we observed tumor and lymph node were remarkably shrunk by CT examination. The patients undergone the cystectomy. In resected specimen, no apparent tumor was observed by pathological examination. The patient did not experience recurrence 2.5 year after radical cystectomy. These results indicated the oncogenic *ERCC2* mutation was a biomarker for predicting patients who respond to platinum-containing therapy.

## Discussion

Comprehensive cancer genome analysis identified significantly mutated genes in several types of cancers. A catalog of cancer-related genes has been established through big efforts (37). Searching selective genes is an effective low-cost approach for identifying somatic mutations. Here, we used a custom-made panel targeting 71 genes and identified at least one mutation in all the tumors examined. We also estimated the functional relevance of each mutation and identified approximately half of the mutations as having oncogenic potential. In this study, we identified an *ERCC2* helicase domain missense mutation (*ERCC2* p.E606Q) in a patient who received platinum-based neoadjuvant therapy. A previous report suggested tumor with *ERCC2* functional deficiency was sensitive to cisplatin (35, 38). As expected, the primary tumor and lymph node metastasis had shrunk after carboplatin-based neoadjuvant therapy. Consistent with this, tumor-derived DNA in urine had also decreased after therapy. However, a patient (Case #14) was not defined as an extreme responder because the patient had residual muscle-invasive disease at cystectomy (pT2a). These differences in drug response is maybe due to the difference of drugs (e.g., cisplatin and carboplatin).

Knowledge databases are important for annotating gene variants. In this study, we used the open-source database OncoKB provided by Memorial Sloan Kettering Cancer Center (27). Thanks to these accumulating datasets, researchers can search the oncogenicity of somatic mutations and assess the driver mutations in tumors. Our data showed that half of the identified mutations had oncogenic potential and most mutations led to a loss of function. The main dysregulated pathways were those involved in chromatin remodeling and epigenetic modifications, as well as the PI3K–AKT–mTOR, p53 cell cycle, and RTK–MAPK pathways, as previously reported (5). We revealed potential drug-matched mutations in 14 out of 19 patients (Table 1). Most matched mutations were in *KDM6A* (*n* = 10), which is likely to be a target of EZH2 inhibitor (39). In the PI3K/AKT pathway, *PIK3CA*-activating mutations were frequently identified (*n* = 7) as well as a *TSC1*-truncating mutation (*n* = 1), which are potentially sensitive to PI3K and mTOR inhibitors, respectively. Though less frequent, *BRAF* (*n* = 1), *BRCA1* (*n* = 1), and *FGFR3* (*n* = 1) mutations were also found. Mismatch repair genes including *MLH1* frameshift (*n* = 1) and *MSH2* splice site (*n* = 1) mutations were identified in two patients, who were thus expected to respond to immune checkpoint inhibitors. Despite the small number of patients in this study, we demonstrated that single agents or combination therapy may be therapeutic options for urothelial BC.

*ERCC2* functions in nucleotide exclusion repair after DNA damage. Recent genomic profiling clarified that *ERCC2* mutations were relatively enriched in 6.7% of urothelial BC compared with several other cancers (27, 28). In this study, we identified a patient harboring an *ERCC2* helicase domain mutation. This mutation, which was glutamic acid (E) to glutamate (Q) at codon 606, was cited in COSMIC and found in urinary tract cancer. Other *ERCC2* mutations, p.E86Q, p.N238S, and p.P463S, were likely to be pathogenic mutations, therefore, other patients would also sensitive to platinum-based therapy. In summary, targeted sequencing analysis could reveal genomic profiles and enhance the determination of optimal therapy for precision medicine.

## Data Availability Statement

The datasets presented in this study can be found in online repositories. The names of the repository/repositories and accession number(s) can be found below: National Bioscience Database Centre (https://biosciencedbc.jp/en/; JGAS00000000241) in JGA database (40).

## Ethics Statement

The studies involving human participants were reviewed and approved by Institutional Review Board of clinical research and genome research committee at Yamanashi Central Hospital. The patients/participants provided their written informed consent to participate in this study.

## Author Contributions

YH designed the study, performed experiments, analyzed data, and wrote the manuscript. HY designed the study, conducted informed consents, and collected samples and clinical data. KA performed laser-capture microdissection and sample preparation. TH and KH conducted informed consents and collected samples and clinical data. TO examined the pathological review of tumor tissues. HM performed data analysis and statistical analysis. MO supervised and designed the study. All authors contributed to the article and approved the submitted version.

## Conflict of Interest

The authors declare that the research was conducted in the absence of any commercial or financial relationships that could be construed as a potential conflict of interest.
